# Residues and Dissipation of the Herbicide Imazapyr after Operational Use in Irrigation Water

**DOI:** 10.3390/ijerph17072421

**Published:** 2020-04-02

**Authors:** Tony M. Dugdale, Kym L. Butler, Mark J. Finlay, Zhiqian Liu, David B. Rees, Daniel Clements

**Affiliations:** 1 Centre for AgriBioscience, Agriculture Victoria, La Trobe University, 5 Ring Rd, Bundoora 3088, Australia; zhiqian.liu@agriculture.vic.gov.au (Z.L.); david.rees@agriculture.vic.gov.au (D.B.R.); daniel.clements@niwa.co.nz (D.C.); 2 Biometrics Team, Hamilton Centre, Agriculture Victoria, 915 Mt Napier Rd, Hamilton 3300, Australia; kym.butler@agriculture.vic.gov.au; 3 Killamont Pty Ltd., Shepparton 3630, Australia; killamont@bigpond.com; 4 National Institute of Water and Atmospheric Research (NIWA), Gate 10 Silverdale Road, Hamilton 3216, New Zealand

**Keywords:** aquatic weed, crop safety, irrigation canal, irrigation channel, *Sagittaria platyphylla*

## Abstract

Imazapyr is a herbicide that can be used in irrigation canals to control a range of aquatic weed species, however, its residual nature, combined with its phytotoxicity to crops at low concentrations, means that the water in canals must be carefully managed following imazapyr application. Residues of the herbicide imazapyr (isopropylamine salt) in irrigation water were analysed and modelled after application to irrigation canals in south-eastern Australia. A treatment program to control delta arrowhead (sagittaria; *Sagittaria platyphylla* (Engelm.) J.G. Sm.) in over 400 km of irrigation canals was enacted by applying imazapyr to dewatered canals during winter. Following imazapyr application, canals were left dewatered for a period (up to eight weeks) and then refilled. After refilling, canals were ponded for a period (up to 28 days) to allow degradation of imazapyr in the water via photolysis. Upon refilling canals, ~650 water samples containing imazapyr were collected across the treatment area and data modelled to measure the extent of water contamination and to guide efforts to reduce the subsequent irrigation hazard to crops. Modelled data demonstrates that imazapyr behaviour in irrigation water following canal refilling was predictable when 1) amount of imazapyr applied, 2) the dewatered period following herbicide application, 3) the water ponding period, and 4) solar exposure during water ponding were taken into account. Minimising the amount applied (g imazapyr per km of canal) and maximising the time between spraying and refilling (dewatered period) reduced the initial concentration in the water following canal refilling. The amount of imazapyr in the canal water following refilling was reduced by half for every 16 days (confidence interval = 10–38 days) that the canal remained dewatered after imazapyr application. Imazapyr dissipation during the ponding period following canal refilling occurred at a rate that depended on solar exposure. Dissipation did not occur when solar exposure was <8.5 MJ m^−2^. However, when solar exposure was >10 MJ m^−2^, imazapyr concentration in the water reduced by half for every 4.4 days of ponding period (confidence interval = 2.9–9.5 days). Our two models, combined with local climate data on solar exposure, can be used by canal managers to determine the optimal time to refill canals so that imazapyr dissipation is maximised, and thus risk of damaging irrigated crops is minimised.

## 1. Introduction

Imazapyr (2-[(RS)-(4-isopropyl-4-methyl-5-oxo-2-imidazolin-2-yl) nicotinic acid) is a systemic, non-selective, pre- and post-emergence herbicide used for the control of a range of aquatic and terrestrial weeds. It prevents the synthesis of the three branched-chain amino acids valine, leucine, and isoleucine by inhibition of the enzyme acetolactate synthase or acetohydroxy acid synthase [1,2]. Imazapyr is soil and foliage active, with plant uptake by roots and leaves. When applied to the soil, it binds and remains active, thus providing residual control. Sorption is considered weak and reversible. The strength varies according to soil characteristics, including pH, organic matter, and clay content, such that it can be highly mobile or persistent in soils [2,3]. Adsorption increases with time and decreases with soil moisture. In terrestrial situations, weed control efficacy persists from three months to two years depending on application rates. Degradation in soil occurs primarily via microbial degradation, with field half-life ranging from 1–5 months depending on soil characteristics and environmental conditions. UV-mediated photolysis (photodegradation) also occurs but is restricted to the soil surface. In water, degradation occurs rapidly via photolysis, with a half-life ranging from 2–5 days [2,4].

In aquatic situations, imazapyr is applied to emergent parts of aquatic weeds and/or the sediment from which they grow. Imazapyr has been used successfully to control aquatic weeds including; common reed (*Phragmites australis* (Cav.) Trin.) [5], smooth cordgrass (*Spartina alterniflora*) [6], melaleuca (*Melaleuca quinquenervia* (Cav.) S.T. Blake), torpedograss (*Panicum repens* L.) [7], and alligator weed (*Alternanthera philoxeroides* (Mart.) Griseb.) [8,9].

In Australia, aquatic weeds obstruct flow of water in irrigation canals reducing their water-carrying capacity and compromising the reliability of water delivery to primary producers [10,11]. The aquatic weed delta arrowhead (sagittaria; *Sagittaria platyphylla* (Engelm.) J.G. Sm.) is particularly abundant in earthen irrigation canals of south-east Australia [12,13]. The herbicide glyphosate is commonly used to control delta arrowhead in this situation. However, high-doses and multiple foliar applications per year are often required to achieve levels of control in which canal hydraulic capacity is not compromised [14,15]. While it might be a relatively safe herbicide from an aquatic environment and irrigated crop toxicology point of view [16], glyphosate’s safety for people is currently a cause for concern [17]. The outcomes of class actions against glyphosate manufacturers create the potential to reduce availability of glyphosate to aquatic weed managers, either as a single active ingredient or when used in combination with other active ingredients (e.g., the propriety combination of glyphosate and imazapyr that is the subject of this article). Various other control methods have been enacted and trialled, generally providing only short-term control [12]. Research is also underway to test suitability of biological control agents to control delta arrowhead [18].

Imazapyr provides an alternative to glyphosate, with similar environmental safety [1,2]. Imazapyr provides a potential control method for delta arrowhead, however, imazapyr is phytotoxic to irrigated pasture and crops at very low rates (the Effective Concentration at which a 25% reduction in biomass occurs (EC_25_) has been reported as low as 0.001 kg (acid equivalent) ae ha^−1^, compared to typical use rates of 0.56 to 1.7 kg ae ha^−1^; [1]). Consequently, a concentration of >1 µg L^−1^ in irrigation water is considered unsafe for irrigation [19]. According to herbicide manufacturers’ label instructions, imazapyr can be used in Australia to control aquatic weeds in irrigation canals. To avoid crop damage, this use-pattern specifies that imazapyr is applied to the exposed sediment of dewatered irrigation canals, and that the canals remain dewatered for a period (dependent on situation) after application. It also specifies that the canals are ponded for a period of time (dependent on situation) after recharging before the water is used for irrigation. The dewatered period after application allows time for the imazapyr to bind to the sediment, and thus reducing the amount of imazapyr released into the water column upon recharge. Upon recharge, imazapyr is expected to remobilise into water via: (i) desorption from sediment (rate varies according to soil properties, temperature, and initial imazapyr binding strength); (ii) entrainment of sediment particles with adsorbed imazapyr (wave action, bioturbation, excavation); (iii) imazapyr release from decaying plants [20]. The ponding period allows time for photolysis to occur thus reducing the amount of imazapyr in the water prior to use for irrigation. It is expected that photolysis occurs with a half-life of 3–5 days [2]. A critical limitation of the use pattern is the requirement to drain canals prior to herbicide application. Therefore, imazapyr has only been applied during the winter irrigation off-season in south-east Australia, which is a ~12-week period between mid-May and mid-August where irrigation water is not delivered [21].

In winter 2015, a program utilising imazapyr to control delta arrowhead in over 400 km of irrigation canals was enacted in Victoria (~36 °S), Australia. A monitoring program following herbicide application determined imazapyr residues in ~650 water samples, from ~200 sites across the treatment area, to ensure residue levels in the water were safe for irrigating crops. This monitoring program showed that imazapyr was present in water samples at the start of the irrigation season, in some situations at levels deemed unacceptable for irrigation (>1 µg L^−1^), resulting in a delayed start to the irrigation season. A flushing program was therefore utilised to flush water containing imazapyr out of the canals before irrigation could proceed. The safe value of 1 µg L^−1^ was derived based on available information [e.g., 1,19], which was considered the level safe for the irrigation of the most sensitive crops. There is uncertainty about how this value relates to flood irrigated crops, as is the case in many parts of Australia, including in this study area, because this value derives from studies where imazapyr was applied with overhead sprinkler irrigation. This uncertainty derives from the absence of published studies determining the phytotoxicity of imazapyr in flood irrigated situations.

Although the program presented difficulties in managing treated water to avoid crop damage, delta arrowhead abundance in canals was reduced substantially, with the area of the infestations <1% of pre-application levels almost two years after imazapyr application (authors’ unpublished data). Based on this information, it appears that the use of imazapyr provides irrigation agencies with a potentially valuable tool to maintain the hydraulic capacity of irrigation canals, if herbicide residues can be managed effectively.

Several excellent studies have been undertaken to understand the fate of herbicides in aquatic environments generally, and irrigation canals particularly, in Australia. These include glyphosate [22,23], dalapon and trichloroacetic acid [24], diuron [25], acrolein [26], dichlobenil [27], and endothall [28]. Many of these studies have been undertaken in canals, while studies available for imazapyr derive from a field-based study in rice paddies in Brazil [17] and several studies in laboratory conditions [29,30,31,32].

To better understand the fate of imazapyr in irrigation canals we examined the relationship between imazapyr residue data collected during the winter 2015 application program and associated operational and environmental data. We present the outcomes of these analyses. Specifically, we present analyses (two general linear models) that relate (1) the concentration of imazapyr in water upon recharging the canals, and (2) the subsequent dissipation of imazapyr in ponded canals, with details of the imazapyr application program, canal dewatering and canal ponding, and how these relationships are affected by a range of climatic variables and canal features. From these relationships, we develop strategic guidelines for minimising the risk of damaging crops irrigated with water taken from irrigation canals where imazapyr has been used to manage aquatic weeds.

## 2. Materials and Methods

### 2.1. Imazapyr Application

Imazapyr was applied to canals, by employees of the statutory authority that manages the canal system (Goulburn-Murray Water), according to the following method:

(1) In May 2015 (late autumn) canals with delta arrowhead infestations were dewatered at the start of the irrigation off-season.
(2) Irrigation canals were then left dewatered for varying periods of time (median 24, min 1, max 60 days before herbicide application).
(3) Imazapyr was then applied as a proprietary product (active ingredient (a.i.): 150 g ae L^−1^ imazapyr (isopropylamine salt) and 150 g ae L^−1^ glyphosate (isopropylamine salt)) at a rate of 750 g imazapyr a.i. ha^−1^ to the foliage of delta arrowhead and the sediment directly below it. This was applied at 5 L ha^−1^ of product with high volume handguns (600 L spray mix ha^−1^) according to herbicide product label instructions. Irrigation canals were treated individually from late May to early July 2015. The total length of canal in this application program was ~400 km.
(4) Irrigation canals were then left dewatered for varying periods of time (median 30, min 8, max 55 days after application).
(5) Irrigation canals were then recharged by filling canals pool by pool, from upstream to downstream (a pool is defined as a length of canal between two flow-regulating structures). Once recharged, pools were left ponded and imazapyr residues in the water were determined at intervals to verify dissipation. Near the end of the irrigation off-season pools with >1 µg L^−1^ imazapyr were flushed to displace imazapyr-contaminated water prior to resumption of supply of irrigation water.

Large scale application of imazapyr by the statutory authority has not occurred since 2015.

### 2.2. Key Environmental Variables in the Region of Imazapyr Application

Canals where herbicide application occurred are constructed of local sediments of the Murray Valley, Central Goulburn and Shepparton Irrigation Districts, which are in alluvial systems influenced by aeolian processes from past drier climates [33,34,35,36]. A range of soils have developed on the alluvial plains but are dominated by texture contrast profiles e.g., light topsoils over heavy subsoils. Soil behaviour of these predominately alkaline soils is characterised by the interaction of calcium carbonate and sodicity of the soils influencing soil structure and drainage. Representative soils are Lemnos loam/Moira loam, Goulburn loam and Boosey loam (Table 1). The irrigation canals supply water by gravity, which infers that the canals are located on naturally high elevation areas, e.g., levee banks, which generally tend to have lighter soil. Sediments, which are likely to be sourced locally (e.g., from canal slumping and scouring), have accrued to varying degrees. Canal efficiency requires regular removal of transported sedimentary material, and sediment distribution is affected by particle size.

The climate of these irrigation districts can be described as having hot dry summers and mild winters. The Koeppen classification is temperate with no dry season and a hot summer [37].

The water for the Murray Valley Irrigation District is sourced from Lake Hume (36° 9.743’S; 147° 3.405’E) while the Central Goulburn and Shepparton Irrigation Districts are supplied by Lake Eildon (37° 6.416’S; 145° 53.976’E). These two reservoirs are supplied from the Eastern Highlands of Victoria. Water in the irrigation canals is typically turbid (40 to 120 Nephelometric Turbidity Units (NTU); Secchi disc depth 17 to 44 cm), fresh (electrical conductivity 50 to 110 µS cm^−1^), neutral (7.5 to 8.5 pH) and has high dissolved oxygen (8.4 to 10.7 mg L^−1^) (Authors’ unpublished data measured in a range of canals in the Central Goulburn and Shepparton Irrigation Districts from September 2017 to March 2018).

The canals used in this study were typically 10 to 20 m wide and 0.5 to 1.5 m deep when at operating level. The lengths of the pools varied, such that the mean volume of the pools was 12.8 megalitres (ML), with a range of 1.5 to 67.4 ML.

### 2.3. Imazapyr Residue Sampling Program and Analytical Method

The water sampling program was determined by operational requirements of the water authority, so that imazapyr residues were <1 µg L^−1^ (crop safety theoretical limit) when irrigation resumed. In total 685 residue water samples from 201 sites were taken. All water samples were filtered using 0.2 µm nylon filter before analysis. One mL of sample, plus 100 μL internal standard in methanol, was analysed using ultra performance liquid chromatography–tandem mass spectrometer (UPLC-MS/MS) AB SCIEX 6500, Zorbax Eclipse Plus C18 rapid resolution high definition (RRHD) column (2.1 × 50 mm, 1.8 μm) with multiple reaction monitoring mode. Imazapyr multiple reaction monitoring (MRM) 262 > 217 was used for quantitation, and MRM 262 > 220 for confirmation. Limit of Detection = 0.0045 µg L^−1^. Limit of Quantitation = 0.008 µg L^−1^. Method error = 0.01 ± 0.003 µg L^−1^. Imazapyr residues were determined by Advanced Analytical Australia Pty Ltd.

### 2.4. Imazapyr Residue Data Collation

To enable examination of imazapyr behaviour, only data from pools of canals that were isolated from upstream imazapyr contamination were used. Many of the water samples collected were taken from sites where contamination from upstream applications of imazapyr occurred. These sites could not be used in the analysis because herbicide treated water and associated concentrations could not be traced back upstream to an initial source due to upstream canal branching.

In total, 16 pools across three irrigation districts (Murray Valley, Shepparton, and Central Goulburn), were isolated and utilised in the analysis. All of these pools had at least one imazapyr sample taken during the ponding period (upon refilling but before flushing) and were used to model the initial imazapyr concentration upon refilling (16 pools). A subset of these pools, where imazapyr samples were taken on at least two separate occasions throughout the ponding period (8 pools), were used to determine imazapyr dissipation during the ponding period. By undertaking these steps we have eliminated any pools where import or export of imazapyr could have occurred, and thus we can be confident that observed changes in the concentration of imazapyr in the pools are due to in-pool dissipation, rather than dilution, loading (by water inflow) or convection (displacement of water associated with flow).

We compiled datasets based on irrigation canal, operational and environmental factors associated with the locations of imazapyr residue sampling including: (1) length, width, and connectedness of irrigation canal pools, (2) flow regulator operation (water flow and water height), (3) imazapyr application records, and (4) weather records (obtained from Australia’s Bureau of Meteorology weather station nearest to each pool). This data was then utilised to model:

1. Initial imazapyr concentration upon canal refilling (Model 1)
2. Dissipation of imazapyr during the ponding period, after canal refilling (Model 2)

### 2.5. Statistical Analysis—Model 1, Initial Imazapyr Concentration

For each flow regulator and its associated pool, the logarithm of the ratio of initial imazapyr concentration on refilling to the amount of imazapyr applied to the canal (including upstream pools) per kilometre was calculated, and denoted ln (RatioIm):

$$\left(ln\left(\frac{\mathrm{initial}\;\mathrm{imazapyr}\;\mathrm{concentration}\;\mathrm{upon}\;\mathrm{refilling}\;\left(\mu g/L\right)}{\mathrm{amount}\;\mathrm{of}\;\mathrm{imazapyr}\;\mathrm{applied}\;\left(g/\mathrm{km}\right)}\right)\right)$$

A general linear model was then developed to relate ln(RatioIm) to pool characteristics, characteristics of herbicide application, local weather conditions and timing of herbicide application, drawdown period, refilling, and initial imazapyr sampling (Table 2). The unit of analysis was a flow regulator and its associated pool.

### 2.6. Statistical Analysis—Model 2, Dissipation of Imazapyr

For each pool, the average relative decay rate of imazapyr between the first and final reading ($\mathrm{ARDC}\%\;=\frac{\int_{t_{0}}^{t_{1}}\frac{1}{I_{t}}\;\frac{dI_{t}}{dt}\;dt}{\int_{t_{0}}^{t_{1}}dt}$; *t*_0_ is time of initial imazapyr sample after filling, *t*_1_ is time of final imazapyr sample and *I_t_* is the concentration of imazapyr at time *t*) was calculated, using the formula (which can be derived analytically):

$$\begin{array}{c} \mathrm{ARDC}\%=100\times \frac{ln\left(\mathrm{initial}\;\mathrm{imazapyr}\;\mathrm{concentration}\;\mathrm{upon}\;\mathrm{refilling}\right)-ln\left(\mathrm{final}\;\mathrm{imazapyr}\;\mathrm{concentration}\right)}{\mathrm{Time}\;\mathrm{between}\;\mathrm{initial}\;\mathrm{and}\;\mathrm{final}\;\mathrm{imazapyr}\;\mathrm{samples}\;(\mathrm{days})}\\ =\frac{100}{\mathrm{Time}\;\mathrm{between}\;\mathrm{initial}\;\mathrm{and}\;\mathrm{final}\;\mathrm{imazapyr}\;\mathrm{samples}\;(\mathrm{days})}\times \\ ln(\frac{\mathrm{initial}\;\mathrm{imazapyr}\;\mathrm{concentration}\;\mathrm{upon}\;\mathrm{refilling}\;}{\mathrm{final}\;\mathrm{imazapyr}\;\mathrm{concentration}\;\mathrm{in}\;\mathrm{refilled}\;\mathrm{pool}\;}) \end{array}$$

A general linear model was then developed to relate ARDC% to pool characteristics, initial imazapyr concentration upon refilling, solar radiation and timing of initial and final imazapyr samplings (Table 3). The unit of analysis was a flow regulator and its associated pool.

## 3. Results

### 3.1. Model 1, Initial Imazapyr Concentration

Characteristics of the pools, the imazapyr application program, and weather variables during the imazapyr application period are shown in Table 2. Critically, average maximum temperatures (12–15 °C) and average solar radiation (6–10 MJ m^−2^) were low, characteristic of temperate winter in northern Victoria, Australia.

**Figure 1:**
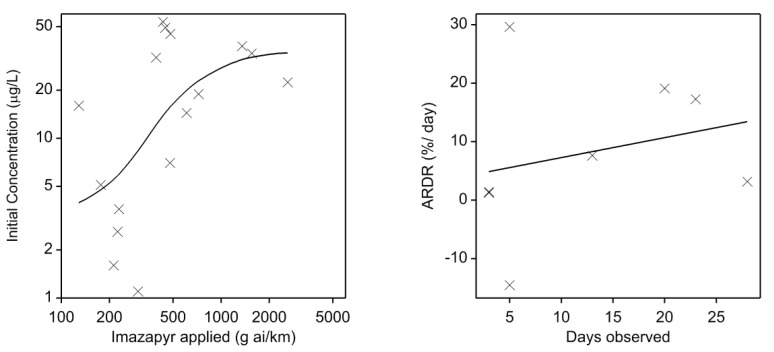
LEFT: Relationship between initial concentration of imazapyr in pools versus imazapyr applied. Each point represents one pool (*n* = 16). Line = smoothing spline of order two. Note log scales. RIGHT: Relationship between percent average relative decay rate (ARDR%) of imazapyr during the ponding period and days observed (i.e., days between first and last sample in the ponding period). Line = smoothing spline of order one.

Imazapyr was applied to patches of delta arrowhead at a rate of 750 g a.i. ha^−1^ over 1 to 41 days (median of one) for each pool. This resulted in application of 129 to 2612 g a.i. km^−1^, with a median of 440 g a.i. km^−1^, applied to the 16 pools (Table 2). Initial imazapyr concentration in the pools following refilling ranged from 1 to 53 µg L^−1^ (median 17; Table 2). The initial imazapyr concentration in the water after refilling the pools was greater for the pools that had more imazapyr applied per unit length (Figure 1).

The logarithm (base e) of the ratio of initial imazapyr concentration upon refilling to the amount of imazapyr applied to the canal (including upstream pools) per kilometre (ln(RatioIm)) was most closely related to the length of the dewatered period after imazapyr application (LengthDW), with longer dewatered period being associated with a lower imazapyr concentration (ln(RatioIm)). The relationship was linear (*p* = 0.0027 on 1, 14 degrees of freedom; variance accounted for = 45%), with no additional quadratic response (*p* = 0.053 on 1, 13 degrees of freedom). There was no evidence (*p* > 0.05) of an effect of any of the other terms in Table 2, once the dewatered period (LengthDW) was included in the model.

The model, excluding the random error, can be written as:

ln(RatioIm) = α −β × LengthDW,

where $\hat{\alpha }$ = −2.37 (s.e. = 0.396) and $\hat{\beta \;\;}$= 0.044 (s.e. = 0.0122)

This can be reparameterised, by exponentiation, as:

RatioIm = Kexp(−β × LengthDW),

where Κ = exp(α).

This equation is in exact accord with constant exponential decay in the amount of imazapyr mobilised from the sediment into the water column, at the time of recharge, and no dissipation of the imazapyr during the early (before the initial imazapyr sampling) ponding period. Κ can be interpreted as the ratio of initial imazapyr concentration upon refilling to the amount of imazapyr applied to the canal (including upstream pools) per kilometre, when the recharge occurs immediately after herbicide application (i.e., when LengthDW = 0). The corresponding half-life of the ‘decay process’ is ln(2) ⁄ β. Κ is estimated as 0.093 (95% confidence interval (CI) = (0.039, 0.218)). The half-life is estimated as 15.6 days (95% CI = (9.8, 38.0)).

Another important consequence of this equation is that it can be rearranged to estimate the final imazapyr concentration in the water column following refilling:

$$\begin{array}{l} Imazapyr\;concentration\;\left(\mu g/L\right)=\\ 0.093\times application\;rate\;\left(g/\mathrm{km}\right)\times 0.957^{\left(days\;of\;dewatering\;post\;application\right)} \end{array}$$

This indicates that the concentration of imazapyr, for a given number of dewatered days after application, is proportional to the amount of imazapyr applied (application rate in g per km of canal). This equation can be used to predict the concentration of imazapyr in the water column upon refilling under a range of scenarios. For example, if 200 g a.i. of imazapyr is applied per km of canal and the canal is refilled immediately the predicted concentration will be 29 µg L^−1^, compared to 1.3 µg L^−1^ if it is left dewatered for 60 days. If 2500 g a.i. per km is applied these figures will be 223 and 17 µg L^−1^, respectively.

In simple terms, solubilisation of imazapyr to water upon refilling occurs at a rate of approximately 10% of that applied, with this value reducing by half for every 16 days of drawdown that refilling is delayed.

### 3.2. Model 2, Dissipation of Imazapyr

Characteristics of the eight pools, imazapyr sampling program and solar exposure during the ponding period are shown in Table 3. The initial imazapyr concentration for the eight pools for which decay data could be calculated ranged from 2 to 54 µg L^−1^ (median of 21), and the number of days over which the decay rate was calculated ranged from 3 to 28 days (median of nine). Critically, average solar exposure was low (8–11 MJ m^−2^), characteristic of temperate late winter in northern Victoria, when day lengths are increasing.

Imazapyr dissipated in some pools but not in others. There was no clear relationship between the number of days that pools were ponded for and relative decay rate (Figure 1).

The average relative decay rate (ARDC%) was most closely related to the average solar exposure between the initial and final imazapyr samplings (AvSolar), with greater solar exposure being associated with a greater ARDC%. The relationship was linear (Probability value (*p*) = 0.0090 on 1, 6 degrees of freedom; variance accounted for = 66%), with no additional quadratic response (*p* = 0.77 on 1, 5 degrees of freedom). There was no evidence (*p* > 0.3) of an effect of any of the other terms in Table 3, once solar exposure was included in the model. A consequence of this is that dissipation of imazapyr in all pools began at approximately the same time, which relates to when solar exposure in the three adjacent irrigation districts had increased.

The model, excluding the random error, can be written as:

ARDC% = α + β × AvSolar,

where $\hat{\alpha }$ = −76 (s.e. = 22.4) and $\hat{\beta \;}$ = 9.2 (s.e. = 2.42)

Another important consequence of this equation is that it can be rearranged to estimate the imazapyr concentration in the water column following refilling after a period of ponding:

$$\begin{array}{l} Final\;imazapyr\;concentration\;\left(\mu g/L\right)=\\ Initial\;imazapyr\;concentration\;\left(\mu g/L\right)\times \exp\left(\left(0.76-0.092\times AvSolar\right)\times Period\right) \end{array}$$

where *Period* is the number of days of ponding.

This indicates that the concentration of imazapyr, for a given number of days of ponding and given average solar radiation, is proportional to the concentration of imazapyr at refilling. This equation can be used to predict how long it will take for imazapyr concentration to fall below 1 µg L^−1^. For example, in late August in northern Victoria (where solar radiation is typically 10 MJ m^−2^) we estimate that it will take 5 days to fall below 1 µg L^−1^ from an initial imazapyr concentration of 2 µg L^−1^, or 26 days if initial concentration was 60 µg L^−1^.

## 4. Discussion

There are three important findings of this study that are useful for waterbody managers. The first is that higher concentrations of imazapyr occur following canal recharge where greater amounts of imazapyr were applied (as mass of active ingredient per km). Although this is intuitive and expected, it provides incentive for managers to take actions that minimise the amount applied. For instance, prudent spot-spraying is likely to be worthwhile, since this will reduce risk of subsequent crop damage.

The second and third important findings are (2) that imazapyr dissipation in water following canal refilling is dependent on solar exposure, which is driven by seasonal progression, and (3) that the reduction in initial imazapyr concentration upon refilling (virtual dissipation), is not affected by environmental conditions. These cannot be manipulated directly by managers, but this knowledge enables us to devise management strategies to obtain the maximum dissipation of imazapyr in the minimum time.

A general strategy is, after application of imazapyr in a dewatered canal during winter conditions, to (i) leave the canal dewatered for a period of time, before (ii) refilling the canal and leaving the canal ponded for a second period of time, so as to maximise the dissipation of imazapyr before the water is released for irrigation. The time that the canal is left dewatered and the time the canal is ponded can be guided by the solar radiation that is expected through the season.

In the case of irrigation areas in northern Victoria, and other irrigation areas in Australia, the irrigation canals are generally not delivering water over a period of several weeks in winter. Therefore, a sensible strategy is to apply imazapyr to dewatered canals over a period early in winter, then leave them dewatered for a period of time in mid-winter when solar radiation is low, and refill the canals later in winter for a period of canal ponding when solar radiation is increasing, before finally releasing the water for irrigation late in winter. In fact, this is what currently occurs (see methods) based on manufacturers’ recommendations (see introduction). However, the results of this study can be used to guide the number of days for which the canal is dewatered, and the number of days for which it is ponded. In particular, we can tabulate half-lives of imazapyr in the two phases as a function of solar radiation (Table 4). Whilst there is quite a deal of variability in our estimates, Table 4 indicates that leaving the canal dewatered provides a shorter imazapyr half-life when average solar radiation is below approximately 9 MJ m^−2^ and ponding the canal provides a shorter imazapyr half-life when average solar radiation is above 9 MJ m^−2^. Certainly, ponding is preferable when average solar radiation is above 10 MJ m^−2^, because at this level the upper confidence limit for the half-life during ponding (9.5 days) is approximately equal to the lowest confidence limit of the half-life during the dewatered period (9.8 days).

The current practice of dewatering canals prior to imazapyr application, leaving canals dewatered for a period after application, and then ponding after refilling to ensure imazapyr is below 1 µg L^−1^ before irrigation, should continue. However, because there is variability within the model that may be associated with individual pool conditions (different locations, depths, sediment properties, water clarity, whether the sample point was near the leading edge of the contaminated water or the trailing edge (which would have substantially less imazapyr)), the half-lives presented in this paper should not be used to prescribe the times at which imazapyr will be < 1 µg L^−1^. These half-lives will be further complicated by the differing amount of imazapyr applied (which is determined by the proportion of the canal occupied by weeds that receive imazapyr application). Therefore, these equations should be used to give guidance on when to change from dewatering to ponding, and then when the most efficient time to start a testing regime to then decide if it is safe to irrigate. Given the substantial cost of determining pesticide residues in water, this may represent considerable cost savings.

These guidelines can help make decisions on the time for transitioning between the dewatered period and ponding period, after application of imazapyr during winter deactivation of canal systems, in various climatic zones. In cool temperate zones, like Atlantic Canada, it would be best to dewater the canal for the whole winter deactivation period. In sub-tropical zones, like Queensland, Australia, it would likely be best to refill the canals and pond water for the winter period. In intermediate temperate zones, like northern Victoria, Australia, it would be best to dewater during the middle of winter and pond water in the later part of winter when solar radiation has increased to 10 MJ m^−2^. Table 4 can be used with knowledge of local climate and weather to guide these decisions.

The findings of this study also highlight the importance of considering if the local conditions are similar to those used in prior testing of pesticide degradation and mobilisation. If pesticides are applied in areas, or at times, that are non-normal then subsequent behaviour may not occur as expected. This is critical for imazapyr due to the large impact of the environment on its dissipation and mobilisation behavior. At the start of the imazapyr-based weed control program described in this study, the waterbody managers assumed that imazapyr would degrade in the water with a 3 to 5-day half-life, based on available literature (summarised in [2] and [4]) and instructions on the product label. Clearly, this did not happen.

There are no published data available on imazapyr use and dissipation in irrigation canals. Rice paddies are a similar shallow aquatic habitat where field studies have been published. Field studies in rice paddies during the growing season have shown that imazapyr dissipation varies according to irrigation regime [38]. The characteristics of the irrigation regime create differing dewatered periods after imazapyr application, water clarity, soil aerobic state, and pH, all of which alter imazapyr dissipation. Dissipation half-lives in paddy water range from 6 to 12 days, with the shorter half-lives being under constant flooding (compared to an intermittent wetting drying cycle). Interestingly, Schreiber et al. [38] suggest that one reason for the shorter half-life in the constant flooding situation was that the water here was clearer, and therefore allowed greater light penetration and photolysis. In another study in rice paddy water, turbidity was identified as a retardant of photolysis of imazethapyr (a related imidazoline herbicide) because of its impact on the availability of sunlight [29]. Our results were also obtained in turbid (~40 to 120 NTU) and shallow (~0.7 m) water but dissipation still occurred with a half-life of 2.3 to 4.4 days, provided solar radiation was above 10 MJ m^−2^.

The half-lives reported in these field studies are in stark contrast to the behavior of imazapyr in some laboratory studies where rapid degradation in water, due to photolysis, occurred under artificial sunlight (half-lives of 1.3 to 2.7 days; [30,31]). However, in a study under natural sunlight, the half-life was 9.1 days [32]. Clearly, the experimental conditions are of critical importance and we believe the statement of Ramezani et al. [32], that predicts abiotic degradation will be slow in the environment and only occur in clear water or on the soil surface, is astute. The discrepancies between different studies underscores the importance of understanding how the local environmental conditions affect pesticide persistence.

Solubilisation and runoff of the herbicides imazapyr, imazethapyr, and imazapic has been shown to be reduced when the period between application to soil in dry rice paddies and subsequent flooding is increased [36,39]. Our findings support these studies and provide an estimate of the magnitude of this effect, i.e., that solubilisation from soil upon flooding is reduced by half for every 16 days between application and flooding.

Based on the considerable variability in imazapyr dissipation under different environmental conditions, we advise that the imazapyr concentration in canals, or any other aquatic environment where it is used, be measured to verify dissipation. For example, deactivation and subsequent mobilisation upon recharge will differ with local soil types, while dissipation will be greatest when water is clear and shallow due to greater penetration of solar radiation into the water.

Although the use of imazapyr described in this study created considerable difficulty associated with protecting irrigated crops, the safety of imazapyr for other sectors of the environment should not be overlooked. For example, the consequences of inadvertent exposure to the environment will be low due to imazapyr’s classification as practically non-toxic to mammals, birds, honeybees, fish, aquatic invertebrates and algae [1]. The risk posed by imazapyr, if it makes its way into rivers and associated aquatic environments, is to aquatic and riparian vegetation, and terrestrial plants, which are all sensitive to this herbicide [1].

## 5. Conclusions

Operational use of imazapyr in irrigation canals shows subsequent concentration in the water after application on sediment was predictable when (1) the amount of imazapyr applied, (2) the dewatered period following imazapyr application, (3) the water ponding period, and (4) solar exposure during water ponding were considered. These quantitative field data characterise the rate of imazapyr dissipation in an aquatic environment in relation to the effect of solar radiation on photolysis and dewatering on sediment sorption. Both processes are important for understanding and managing imazapyr residues in the environment and both have scant data derived from aquatic environments. The concentration of imazapyr in the water following refilling was reduced by half for every 16 days that the canal remained dewatered after imazapyr application. After canal refilling, imazapyr dissipation reliably occurred when solar exposure was >10 MJ m^−2^, but little dissipation occurred when it was lower than this. Information is presented that can be used to determine an application and ponding program that ensures imazapyr levels are safe before the water is used for irrigation. This study is limited by the environment in which it was undertaken. Thus, these models will require local verification as mobilisation is likely to be different with different soil types and dissipation will differ with different water depth and water clarity. Future studies to validate our findings in different situations and geographic locations will provide an improved understanding of imazapyr dissipation in surface waters.

## Figures and Tables

**Table 1:** Classification of representative soils (three most numerous) in the irrigation districts used in this study.

Soil Name	Proportion of Irrigation Area (%)	Australian Soil Classification (ASC)	World Reference Base for Soil Resources (WRB)
Lemnos loam/Moira loam	40	Red Sodosol/Chromosol	Luvisol, Solonetz
Moira loam/Goulburn loam	25	Brown Sodosols	Solonetz
Goulburn Boosey loam	10	Grey Sodosol/Vertosol	Solonetx, Vertisol

**Table 2:** Terms assessed for inclusion in the model for predicting the ratio of the first imazapyr concentration recorded after the start of ponding to the amount of imazapyr applied per km of canal (Model 1—Initial imazapyr concentration). Ln denotes logarithm to base e.

Term	Median	Min.	Max.	Comments
*Pool characteristics*				
Irrigation district	3 level factor			Murray Valley, Shepparton, and Central Goulburn
Canal name	9 level factor			Canals MVS1, SPS1, SPS2, SPS3, RNIS1, RNIS2, MVIS7, SPIS8, and RNIS9
Dead end spur indicator	2 level factor			Yes or no, depending if canal was a dead-end spur
*Variables*				
Length of canal treated (excluding upstream; km)	1.4	0.3	7.9	
Length of canal treated (including upstream; km)	5.5	0.7	13.4	
Amount of imazapyr applied (excluding upstream; g km^−1^)	331	23	2612	
Amount of imazapyr applied (including upstream; g km^−1^)	440	129	2612	
Initial concentration (µg L^−1^)	17	1	53	
Days after spraying until recharge (dewatered period)	30	8	55	
Number of days between start and end of application (excluding upstream)	1	0	40	
Number of days between start and end of application (including upstream)	7	1	40	
Days drawdown period after application	30	8	55	
Total imazapyr (g a.i.) applied excluding upstream	548	14	5060	
Total imazapyr (g a.i.) applied including upstream	1952	253	9688	
Volume (ML) to recharge pool excluding upstream	6.4	1.5	67.4	
Days drawdown before application	24	1	60	
Days with rainfall before application including upstream	7	0	20	
Days with rainfall during application including upstream	0	0	6	
Days with rainfall during drawdown after application	8	0	11	
Days with rainfall during drawdown before application excluding upstream	8	0	11	
Days with rainfall during drawdown and during application excluding upstream	0	0	6	
Days with rainfall during drawdown after application including upstream	0	0	6	
Rainfall (mm) during drawdown after application excluding upstream	38	1	61	
Rainfall (mm) during drawdown before application excluding upstream	15	0	56	
Rainfall (mm) during drawdown and during application excluding upstream	0	0	37	
Rainfall (mm) during drawdown after application including upstream	38	1	61	
Rainfall (mm) during drawdown before application including upstream	15	0	56	
Rainfall (mm) during drawdown and during application including upstream	0	0	37	
Solar exposure (MJ m^−2^) during drawdown after application excluding upstream	8.1	7.4	8.6	
Solar exposure (MJ m^−2^) during drawdown before application excluding upstream	8.5	7.8	11.1	
Solar exposure (MJ m^−2^) during drawdown and during application excluding upstream	7.8	4.5	9.7	
Solar exposure (MJ m^−2^) during drawdown after application including upstream	8.1	7.4	8.6	
Solar exposure (MJ m^−2^) during drawdown before application including upstream	8.5	7.8	11.1	
Solar exposure (MJ m^−2^) during drawdown and during application including upstream	7.8	4.5	9.7	
Air temperature (℃) during drawdown after application excluding upstream	12.9	12.3	15.0	
Air temperature (℃) during drawdown before application excluding upstream	15.2	14.4	21.1	
Air temperature (℃) during drawdown and during application excluding upstream	13.9	11.8	20.4	
Air temperature (℃) during drawdown after application including upstream	12.9	12.3	15.0	
Air temperature (℃) during drawdown before application including upstream	15.2	14.4	21.1	
Air temperature (℃) during drawdown and during application including upstream	13.9	11.8	21.1	
Recharge to first sample (days) during ponding excluding upstream	8	1	44	
Recharge to first sample (days) during ponding including upstream	8	1	44	
Solar exposure (MJ m^−2^) during recharge excluding upstream	8.2	6.8	9.2	
Solar exposure (MJ m^−2^) during recharge including upstream	8.2	6.8	9.2	
Recharge start date	29 July	3 July	5 August	Included as number of days since start of year
Herbicide application end date	30 June	20 May	20 July	
Herbicide application start date	10 June	19 May	10 July	

**Table 3:** Terms assessed for inclusion in the model of imazapyr dissipation during ponding (Model 2).

Term	Median	Min.	Max.	Comments
*Pool characteristics*				
Irrigation district	3 level factor			Murray Valley, Shepparton, and Central Goulburn
Canal name	9 level factor			Canals MVS1, SPS1, RNIS1, RNIS2 and RNIS9
Dead end spur indicator	2 level factor			Yes or no, depending if canal was a dead-end spur
*Variables*				
Concentration of initial sample (µg L^−1^)	20.7	2.2	53.5	
Average solar exposure during observation period (AvSolar; MJ m^−2^)	8.6	8	11.4	
Solar exposure (MJ m^−2^) in first 5 days following initial sample (or up to final sample if less than 5 days ponding period)	8.2	7.9	11.4	
Days between initial and final sample	9	3	28	
Date of initial sample (days after 1/7/15)	35	12	55	
Date of final sample (days after 1/7/15)	45	15	62	
Estimated water depth (cm)	74	57	108	
Estimated sediment depth (cm)	46	16	108	

**Table 4:** Days to dissipate imazapyr in the water column and days to “dissipation” in the dewatered period for different levels of average solar radiation during the ponding period. LCL = Lower 95% confidence limit; UCL = Upper 95% confidence limit.

Average Solar Radiation (MJ m^−2^)	Half-Life during Dewatered Period (Days)			Half-Life during Ponding (Days)		
	Estimate	LCL	UCL	Estimate	LCL	UCL
8	15.6	9.8	38.0	No dissipation ^a^	9.7	No dissipation ^a^
8.5	15.6	9.8	38.0	35.6	7.0	No dissipation ^a^
9	15.6	9.8	38.0	10.6	5.1	No dissipation ^a^
9.5	15.6	9.8	38.0	6.2	3.8	17.4
10	15.6	9.8	38.0	4.4	2.9	9.5
10.5	15.6	9.8	38.0	3.4	2.3	7.0
11	15.6	9.8	38.0	2.8	1.8	5.7
11.5	15.6	9.8	38.0	2.3	1.5	4.9

^a^ = best estimate (or confidence limit) of β is a negative value, indicating that dissipation will not occur.

## References

[1] Imazapyr—Human Health and Ecological Risk Assessment–Final Report. https://www.fs.fed.us/foresthealth/pesticide/pdfs/Imazapyr_TR-052-29-03a.pdf.

[2] Shaner D.L., Weed Science Society of America (WSSA) (2014). Imazapyr. Herbicide Handbook.

[3] Ecological Risk Assessment of the Proposed Use of the Herbicide Imazapyr to Control Invasive Cordgrass (*Spartina* spp.) in Estuarine Habitat of Washington State. http://www.ecy.wa.gov/programs/wq/pesticides/final_pesticide_permits/noxious/risk_assessment_Imazapyr.pdf.

[4] United States Environmental Protection Agency (USEPA) (2006). Reregistration Eligibility Decision (RED) for Imazapyr.

[5] Kay S.H. (1995). Efficacy of wipe-on applications of glyphosate and imazapyr on common reed in aquatic sites. J. Aquat. Plant Manag..

[6] Patten K. (2002). Smooth cordgrass (*Spartina alterniflora*) control with imazapyr. Weed Tech..

[7] Netherland M.D., Getsinger K.D., Stubbs D.R. (2005). Aquatic plant management: Invasive species and chemical control. Outlook Pest Manag..

[8] Clements D., Dugdale T.M., Butler K.L., Florentine S.K., Sillitoe J. (2017). Herbicide efficacy for aquatic *Alternanthera philoxeroides* management in an early stage of invasion: Integrating above-ground biomass, belowground biomass and viable stem fragmentation. Weed Res..

[9] Dugdale T.M., Champion P.D. (2012). Control of alligator weed with herbicides: A review. Plant Prot. Q..

[10] Clements D., Butler K.L., Hunt T.D., Liu Z., Dugdale T.M. (2018). Efficacy of endothall dimethylalkylamine salt applied to static irrigation channels during winter to control aquatic weeds in temperate Australia. J. Aquat. Plant Manag..

[11] Dugdale T.M., Hunt T.D., Clements D. (2013). Aquatic weeds in Victoria: Where and why are they a problem, and how are they being controlled?. Plant Prot. Q..

[12] Clements D., Dugdale T.M., Butler K.L., Hunt T. (2015). Control of delta arrowhead (*Sagittaria platyphylla*) in Australian irrigation channels with long exposure to endothall dipotassium salt during winter. J. Aquat. Plant Manag..

[13] Kwong R.M., Sagliocco J.L., Harms N.E., Butler K.L., Green P.T., Martin G.D. (2017). Biogeographical comparison of the emergent macrophyte, *Sagittaria platyphylla* in its native and introduced ranges. Aquat. Bot..

[14] Adair R.J., Keener B.R., Kwong R.M., Sagliocco J.L., Flower G.E. (2012). The biology of Australian weeds, 60: *Sagittaria platyphylla* (Engelmann) J.G. Smith and S. Calycina Engelmann. Plant Prot. Q..

[15] Developing Best Practice Management Strategies for Sagittaria in Australia. Phase 1: Current Management Practices—May 2018. http://www.riverinaweeds.org.au/wp-content/uploads/2018/06/Developing-best-practice-management-strategies-for-sagittaria-in-Australia-May-2018-FINAL.pdf.

[16] Solomon K., Thompson D. (2003). Ecological risk assessment for aquatic organisms from over-water uses of glyphosate. J. Toxicol. Environ. Health Part B.

[17] Zhang L., Rana I., Shaffer R.M., Taioli E., Sheppard L. (2019). Exposure to glyphosate-based herbicides and risk for non-Hodgkin lymphoma: A meta-analysis and supporting evidence. Mutat. Res. Rev. Mutat. Res..

[18] Kwong R.M., Broadhurst L.M., Keener B.R., Coetzee J.A., Knerr N., Martin G.D. (2017). Genetic analysis of native and introduced populations of the aquatic weed *Sagittaria platyphylla*–implications for biological control in Australia and South Africa. Biol. Control.

[19] Massachusetts Department of Agricultural Resources (MDAR) (2012). Imazapyr: Review for Use in Lakes & Ponds in Massachusetts. http://www.mass.gov/eea/docs/agr/pesticides/aquatic/imazapyr.pdf.

[20] Tu M., Hurd C., Randall J.M. (2001). Weed Control Methods Handbook: Tools and Techniques for Use in Natural Areas. The Nature Conservancy. http://tncweeds.ucdavis.edu/handbook.html.

[21] Clements D., Dugdale T.M., Hunt T.D. (2013). Determining the efficacy of the herbicides endothal and diquat on the aquatic weed sagittaria in irrigation channels. Plant Prot. Q..

[22] Bowmer K.H. (1982). Residues of glyphosate in irrigation water. Pestic. Sci..

[23] Bowmer K.H., Michael P., Boulton D., Short D.L., Higgins M.L. (1986). Glyphosate–sediment interactions and phytotoxicity in turbid water. Pestic. Sci..

[24] Bowmer K.H. (1987). Residues of dalapon and TCA in sediments and irrigation water. Pestic. Sci..

[25] Bowmer K.H., Adeney J.A. (1978). Residues of diuron and phytotoxic degradation products in aquatic situations. II. Diuron in irrigation water. Pestic. Sci..

[26] Bowmer K.H., Higgins M.L. (1977). Some aspects of the persistence and fate of acrolein herbicide in water. Arch. Environ. Contam. Toxicol..

[27] Bowmer K.H., O’Loughlin E.M., Shaw K., Sainty G.R. (1976). Residues of dichlobenil in irrigation water. J. Environ. Qual..

[28] Islam M.S., Hunt T.D., Liu Z., Butler K.L., Dugdale T.M. (2018). Sediment facilitates microbial degradation of the herbicides endothall monoamine salt and endothall dipotassium salt in an aquatic environment. Int. J. Environ. Res. Public Health.

[29] Avila L.A., Massey J.H., Senseman S.A., Armbrust K.L., Lancaster S.R., Mccauley G.N., Chandler J.M. (2006). Imazethapyr aqueous photolysis, reaction quantum yield, and hydroxyl radical rate constant. J. Agric. Food Chem..

[30] Mangels G., Shaner D.L., O’Connor S.L. (1991). Behavior of the Imidazolinone herbicides in the aquatic environment. The Imidazolinone Herbicides.

[31] Mallipudi N.M., Stout S.J., DaCunha A.R., Lee A.H. (1991). Photolysis of imazapyr (AC 243997) herbicide in aqueous media. J. Agric. Food Chem..

[32] Ramezani M., Oliver D.P., Kookana R.S., Gill G., Preston C. (2008). Abiotic degradation (photodegradation and hydrolysis) of imidazolinone herbicides. J. Environ. Sci. Health Part B.

[33] Butler B.E., Baldwin J.W., Penman F., Downes G.W. (1942). Soil Survey of Part of the County of Moira, Victoria.

[34] Johnston E.J. (1952). The Soils of the Western Part of the Murray Valley Irrigation Area.

[35] Skene J.K.M., Poutsma T.J. (1962). Soils and Land Use in Part of the Goulburn Valley, Victoria.

[36] Skene J.K.M. (1963). Soils and Land Use in the Deakin Irrigation Area, Victoria.

[37] Bureau of Meteorology (2016). Australian Government Bureau of Meteorology Climate Classification Maps. http://www.bom.gov.au/jsp/ncc/climate_averages/climate-classifications/index.jsp?maptype=kpn#maps.

[38] Schreiber F., Scherner A., Massey J.H., Zanella R., Avila L.A. (2017). Dissipation of clomazone, imazapyr, and imazapic herbicides in paddy water under two rice flood management regimes. Weed Tech..

[39] Martini L.F.D., Mezzomo R.F., de Avila L.A., Massey J.H., Marchesan E., Zanella R., Peixoto S.C., Refatti J.P., Cassol G.V., Marques M. (2013). Imazethapyr and imazapic runoff under continuous and intermittent irrigation of paddy rice. Agric. Water Manag..

